# Integrative Oncology

**DOI:** 10.1155/2013/124032

**Published:** 2013-12-19

**Authors:** Gustav Dobos, Holger Cramer, Shrikant Anant, Claudia M. Witt, Lorenzo Cohen

**Affiliations:** ^1^Department of Internal and Integrative Medicine, Kliniken Essen-Mitte, Faculty of Medicine, University of Duisburg-Essen, 45276 Essen, Germany; ^2^University of Kansas Cancer Center, Kansas City, KS 66160, USA; ^3^Institute for Social Medicine, Epidemiology, and Health Economics, Charité Universitätsmedizin Berlin, 10098 Berlin, Germany; ^4^Center for Integrative Medicine, Maryland School of Medicine, University of Maryland, Baltimore, MD 21201, USA; ^5^Department of General Oncology and the Integrative Medicine Program, The University of Texas MD Anderson Cancer Center, Houston, TX 77030, USA

Complementary treatment approaches are increasingly requested by cancer patients and survivors to reduce side effects of conventional cancer treatment, enhance health-related quality of life, and improve clinical outcomes [1]. As many patients do not disclose their complementary therapies use to their oncologist, treatment plans are often not coordinated [2]. Thus, the risk of adverse events and interactions of complementary with conventional therapies is increased. An informed alliance of conventional and complementary treatment providers is important to optimize health outcomes and safety. Integrative oncology that combines conventional cancer treatment with evidence-based complementary therapies and mind/body medicine has become a growing field of health care that strives to address the limitations inherent in fractionated medical care. In integrative oncology, conventional and complementary approaches are combined in order to optimize outcomes and prevent adverse events [3].

While the evidence for both conventional and complementary cancer treatments is increasing, few studies have explicitly tested an integrative oncology approach. The publication of the Society for integrative oncology practice guidelines in 2007 (updated in 2009) has been an important milestone [4, 5]; however, more research is needed that investigates the effectiveness, safety, and optimal design of integrative oncology interventions.

This special issue is dedicated to research in the field of integrative oncology. The peer-reviewed issue solicited original manuscripts in the field of integrative oncology with a focus on studies that combine conventional and complementary approaches to optimize patients' health outcomes. The accepted manuscripts represent a broad range of research including basic, clinical research, qualitative, and survey studies. Six of the included papers present the results of in vitro studies of herbs or natural products from Traditional Chinese Medicine, Traditional Korean Medicine, and Traditional African Medicine in animal or human cancer cell lines. One of these in vitro studies explicitly adopted an integrative point of view in that it investigated the synergistic inhibition of angiogenesis by the Chinese herb Artesunate and the western ACE inhibitor Captopril.

Two further papers investigate the use of complementary and alternative medicine in pediatric oncology. A survey among Canadian pediatric cancer patients found that almost two-thirds had used complementary medicine. Another paper evaluated pediatric oncology best-cases based on NCI criteria. Complementary therapies use was also investigated in another survey in adult US breast cancer patients, focusing specifically on self-care approaches. More than two-thirds of the sample used complementary approaches. In a qualitative study, the needs and concerns of women during breast cancer chemotherapy and the perceived benefits of acupuncture were investigated. In this study, a highly valued outcome was enabling coping through the alleviation of symptoms and increased well-being.

The remaining two papers explicitly addressed implementation of integrative oncology models in clinical practice. In a qualitative study, integration models in integrative oncology centers in Germany and the USA were investigated. Although differing in their type of integration model, this study found similarities regarding the philosophies and priorities between clinics. Both clinics placed an emphasis on research and evidence with the goal to offer evidence-based treatments. Finally, a review of integrative oncology discusses important theoretical, practical, and research issues.

We are certain that this special issue, that covers a spectrum of Integrative Oncology, will advance the evidence-base of this emerging field and inform practitioners and researchers alike.
